# New Bounds on the Aperiodic Hamming Correlation of Wide-Gap Frequency-Hopping Sequence Sets

**DOI:** 10.3390/e28101094

**Published:** 2026-10-08

**Authors:** Peihua Li

**Affiliations:** Sichuan Internet College, Sichuan Normal University, Chengdu 610101, China; peihuali@sicnu.edu.cn

**Keywords:** wide-gap frequency-hopping sequence sets, aperiodic hamming correlation, Peng–Fan lower bound, frequency-hopping multiple access, anti-jamming communications, sequence design

## Abstract

Wide-gap frequency-hopping sequences (WGFHSs) require a sufficiently large frequency gap between adjacent frequency points, which grants them inherent resistance against tracking jamming, barrage jamming, and multipath fading. Such sequences constitute a critical building block for enhancing the anti-jamming performance of frequency-hopping communication systems operating in complex electromagnetic environments. In practical systems, the aperiodic Hamming correlation of sequences directly affects receiver synchronization and sequence acquisition performance. Hence it bears greater practical significance than periodic Hamming correlation. However, owing to the complexity of application scenarios, related research is challenging, and existing results remain limited. In this paper, we extend the traditional Peng-Fan aperiodic bound to WGFHSs, and derive two lower bounds on the maximum aperiodic Hamming correlation of WGFHS sets. Numerical examples demonstrate that the proposed bounds are tighter than the traditional Peng–Fan aperiodic bound. This provides a more rigorous theoretical foundation for the design of WGFHS sets.

## 1. Introduction

Frequency-hopping multiple-access (FHMA) spread-spectrum communication employs information-independent pseudorandom sequences to expand the radio-frequency signal bandwidth far beyond the baseband signal bandwidth. Spread-spectrum processing reduces the power spectral density of transmitted signals, enabling reliable communication at low signal-to-noise ratios. At the receiver, despreading yields a processing gain, thereby improving the anti-interference tolerance of the system. Owing to its prominent advantages including strong anti-jamming, anti-interception, and multi-user networking capabilities [1], it has been widely applied in various wireless communication scenarios that simultaneously support voice and data transmission, including military anti-jamming communications, Bluetooth, and smart grids (see Refs. [2,3,4,5], and the references therein). In frequency-hopping communication systems, the carrier frequencies of each user are governed by a frequency-hopping sequence (FHS). The multiple-access interference among different users is directly determined by the Hamming correlation properties of FHSs: the auto-correlation characterizes the self-interference generated by the same user under different time delays, while the cross-correlation describes the mutual interference between distinct users [6,7]. Therefore, the design of FHSs with excellent Hamming correlation performance is crucial to guaranteeing the communication quality of frequency-hopping communication systems, and various theoretical bounds on Hamming correlation provide important theoretical criteria for evaluating the performance and guiding the construction of FHSs.

In fact, most existing studies on FHSs concentrate on periodic Hamming correlation (see Refs. [6,7,8,9,10]). However, in practical engineering applications, owing to limited synchronization time, the effective observation window of the signal at the receiver is usually shorter than the full sequence period [11]. In other words, aperiodic correlation describes the interference conditions within a truncated observation window, and such interference cannot be fully captured by periodic correlation metrics. Therefore, the aperiodic Hamming correlation can more realistically characterize the interference level in actual communication processes and exhibit higher engineering reference value for system performance evaluation. A larger aperiodic Hamming correlation value indicates more frequency collisions between the target signal and interferers or unintended multi-user interference, which further aggravates link interference and increases the packet loss probability. In contrast, a smaller correlation value implies fewer frequency collisions and thus superior anti-interference capability of the system. The well-known Lempel-Greenberger bound gives a theoretical lower bound on the maximum periodic Hamming auto-correlation of a single FHS. On this basis, the Peng-Fan bound is further extended to establish the lower bound of the maximum periodic Hamming correlation for FHS sets, which has become a classical theoretical achievement in the field of periodic Hamming correlation for FHSs. For aperiodic application scenarios, the Peng-Fan aperiodic bounds provide the theoretical lower bounds on the maximum aperiodic Hamming correlation of FHS sets, which serve as an important criterion for evaluating the aperiodic Hamming correlation performance of FHSs [7]. However, conventional FHSs impose no constraints on the adjacent frequency gap. If the frequency gap between consecutive frequency points is small, the system becomes highly susceptible to single-frequency narrow-band interference, partial-band jamming, tracking jamming, and multipath fading. Once a certain frequency-hopping signal falls into the interference-occupied frequency band, its adjacent frequency signals are also prone to being jam-covered, which renders it difficult for the receiver to recover the corrupted information through channel coding. This is particularly harmful in tracking jamming and partial-band jamming environments commonly encountered in radar, Bluetooth, and millimeter-wave systems.

To mitigate the aforementioned engineering challenges, the wide-gap frequency-hopping sequences (WGFHSs) have been proposed [12,13], and the core constraint is that the difference between adjacent frequency points of the sequence must exceed a preset minimum gap. This inherent property constitutes a core advantage of WGFHS over conventional FH in single-frequency, narrowband, and partial-band interference scenarios. It helps suppress frequency collisions and thereby enhances the anti-interference capability of the system. This class of sequences was first developed in the Joint Tactical Information Distribution System (JTIDS), and can effectively resist narrow-band interference, band-blocking jamming and tracking jamming, thus holding significant application value in the field of anti-jamming wireless communications [14,15]. It is worth noting that, despite some advances in the study of WGFHSs, the existing theoretical bounds predominantly target the periodic Hamming correlation, and research pertaining to the aperiodic Hamming correlation bounds for WGFHSs is still severely insufficient. Li et al. generalized the Lempel-Greenberger bound and the Peng-Fan periodic bound to derive the lower bound for periodic Hamming correlation of WGFHSs, and further investigated the partial Hamming correlation bounds for such WGFHSs [16,17]. In 2025, Tian et al. derived the aperiodic bounds for WGFHSs [18]. Subsequently, Yang and Zheng et al. separately investigated the lower bounds on the aperiodic Hamming correlation of WGFHS sets under low-hit-zone and no-hit-zone constraints [19,20]. These lower bounds are specifically designed for quasi-synchronous FHMA systems to limit the range of aperiodic Hamming correlation within a predetermined time-delay window. In 2026, Wen et al. investigated the periodic and partial Hamming correlation bounds for multi-slot WGFHSs [21].

As emphasized above, the aperiodic Hamming correlation is more indicative of the actual anti-jamming capability of a system in practical communications than its periodic counterpart. Since the traditional Peng–Fan aperiodic bound lacks the wide-gap constraint, its direct application to the performance assessment of WGFHS sets yields bounds that are too loose to precisely capture the parametric restrictions imposed by the wide-gap requirement on aperiodic correlation performance. This deficiency restricts both the deployment and the performance evaluation of WGFHSs in practical systems. Consequently, the derivation of aperiodic Hamming correlation bounds for WGFHS sets possesses both important theoretical significance and urgent engineering application value.

Motivated by this research gap, this paper investigates the aperiodic Hamming correlation bounds for WGFHS sets. By extending the traditional Peng–Fan aperiodic bound to WGFHS sets, we derive two lower bounds on the maximum aperiodic Hamming correlation of WGFHS sets. Furthermore, numerical examples verify that the proposed bounds are tighter than the traditional Peng–Fan aperiodic bounds, thereby enabling a more precise characterization of the theoretical limit of the aperiodic Hamming correlation for WGFHS sets. The findings of this paper enrich the theoretical framework of WGFHSs and can provide theoretical support for the optimal construction and performance evaluation of WGFHS sets with respect to aperiodic Hamming correlation.

The remainder of this paper is organized as follows. In Section 2, we will review some preliminaries. In Section 3, we will derive two lower bounds on aperiodic Hamming correlation for WGFHS sets. In Section 4, we will provide numerical examples to verify the tightness and attainability of the proposed bounds, together with a comparison against the traditional Peng-Fan aperiodic bounds and the aperiodic WGFHS bounds. Finally, Section 5 concludes the paper.

## 2. Preliminaries

In this section, we will review several fundamental definitions concerning FHSs, including the aperiodic Hamming correlation function and wide-gap frequency-hopping sequences.

### 2.1. Aperiodic Hamming Correlation Function

Throughout this paper, let $F=\{f_{0},f_{1},\cdots ,f_{q-1}\}$ denote an alphabet of size *q*, where $f_{i}$ is a frequency, and *q* is a positive integer. Generally, F is defined by a set of integers {0,1,⋯,q−1}.

**Definition** **1** ([6])**.** *Let $X=(x_{0},x_{1},\cdots ,x_{N-1})$ and $Y=(y_{0},y_{1},\cdots ,y_{N-1})$ be any two FHSs of period N over alphabet F. The periodic Hamming correlation of FHSs X and Y at a shift τ is defined by*

(1) $$\begin{array}{c} H_{X,Y}\left(\tau \right)=\sum_{i=0}^{N-1}h\left[x_{i},y_{i+\tau }\right],\;\;0\leq \tau <N, \end{array}$$

*where the modulo N is performed to calculate the index i+τ, and h[a,b]=0 if a≠b and* 1 *otherwise.*

In particular, when X=Y, $H_{X,X}\left(\tau \right)$ is referred to as the periodic Hamming auto-correlation function, which represents the number of frequency collisions between the sequence *X* and itself within one sequence period at a relative time delay τ, and is simply denoted by $H_{X}\left(\tau \right)$. When X≠Y, $H_{X,Y}\left(\tau \right)$ is referred to as the periodic Hamming cross-correlation function, which represents the number of frequency collisions between the sequences *X* and *Y* within one sequence period at a relative time delay τ. Obviously, a smaller value of $H_{X,Y}\left(\tau \right)$ corresponds to fewer collisions between the two sequences, and hence lower mutual interference.

**Definition** **2** ([7])**.** *The aperiodic Hamming correlation of FHSs X and Y at a shift τ is defined by*

(2) $$\begin{array}{c} A_{X,Y}\left(\tau \right)=\sum_{i=0}^{N-1-\tau }h\left[x_{i},y_{i+\tau }\right],\;\;0\leq \tau <N. \end{array}$$

Similarly, when X=Y, $A_{X,X}\left(\tau \right)$ is called the aperiodic Hamming auto-correlation function, abbreviated as $A_{X}\left(\tau \right)$. When X≠Y, $A_{X,Y}\left(\tau \right)$ is referred to as the aperiodic Hamming cross-correlation function.

**Definition** **3.** *Let S be a set of M FHSs of period N over an alphabet F of size q. The maximum aperiodic Hamming auto-correlation function $A_{a}\left(X\right)$ for any single FHS X in S, the maximum aperiodic Hamming auto-correlation (AHAC) function $A_{a}\left(S\right)$ of S, the maximum aperiodic Hamming cross-correlation (AHCC) function $A_{c}\left(S\right)$ of S, and the maximum aperiodic Hamming correlation (AHC) function $A_{m}\left(S\right)$ of S are defined respectively as follows:*

$$\begin{array}{cc} A_{a}\left(X\right) & =\max\{A_{X}\left(\tau \right)|0<\tau <N\},\\ A_{a}\left(S\right) & =\max\{A_{X}\left(\tau \right)|X\in S,0<\tau <N\},\\ A_{c}\left(S\right) & =\max\{A_{X,Y}\left(\tau \right)|X,Y\in S,X\neq Y,0\leq \tau <N\},\\ A_{m}\left(S\right) & =\max\{A_{a}\left(S\right),A_{c}\left(S\right)\}. \end{array}$$

For brevity, we write $A_{a}=A_{a}\left(S\right)$, $A_{c}=A_{c}\left(S\right)$ and $A_{m}=A_{m}\left(S\right)$ in the subsequent sections. A single sequence *X* is called an $\left(N,q;A_{a}\left(X\right)\right)$-FHS, and the set S is called an $\left(N,M,q;A_{m}\left(S\right)\right)$-FHS set.

In 2004, Peng and Fan derived the following lower bound on the maximum AHC $A_{m}$ of FHS sets, which serves as one of the important metrics for evaluating the aperiodic Hamming correlation performance of FHS sets.

**Lemma** **1** (Peng-Fan aperiodic bounds [7])**.** *Let S be an $\left(N,M,q;A_{m}\right)$-FHS set, then*

(3) $$\begin{array}{cc} A_{m} & \geq \left\lceil \frac{(3MN-Nq-N-M-q)N}{\left(2MN-M-1)(M+q\right)}\right\rceil , \end{array}$$

*and*

(4) $$\begin{array}{cc} A_{m} & \geq \left\lceil \frac{\left(4I-N-1)MN-I(I+1)(M+q\right)}{(2MN-M-1)M}\right\rceil , \end{array}$$

*where I=⌊2MN/(M+q)⌋.*

It is claimed that the set S is said to be optimal with respect to the aperiodic Hamming correlation if its relevant parameters achieve the equal sign of Equation (3) or Equation (4).

### 2.2. Wide-Gap Frequency-Hopping Sequences

**Definition** **4** ([12,14])**.** *Let S be a set of M FHSs with period N over alphabet F of size q. For any FHS $s_{i}=\left(s_{i}\left(0\right),s_{i}\left(1\right),\ldots ,s_{i}\left(N-1\right)\right)$ in S, where 0≤i≤M−1, define*

$$\begin{array}{cc} d_{i} & =\min_{0\leq t\leq N-2}\left\{\right|s_{i}\left(t+1\right)-s_{i}\left(t\right)|,|s_{i}\left(N-1\right)-s_{i}\left(0\right)\left|\right\}-1. \end{array}$$

*If $d_{i}>0$, then $s_{i}$ is called a wide-gap frequency-hopping sequence (WGFHS) with minimum gap $d_{i}$. For the sequence set S, define*

$$\begin{array}{cc} D & =\min_{0\leq i\leq M-1}\left\{d_{i}\right\}. \end{array}$$

*If D>0, then S is called a WGFHS set with minimum gap D.*

In 2025, Tian et al. derived the following bounds on the maximum AHC $A_{m}$ of WGFHS sets.

**Lemma** **2** ([18])**.** *Let S be an $\left(N,M,q;A_{m}\right)$-WGFHS set, then*

(5) $$\begin{array}{cc} A_{m} & \geq \left\lceil \frac{3MN^{2}-MN-qN^{2}-q}{\left(2MN-M-2)(M+q\right)}\right\rceil , \end{array}$$

*and*

(6) $$\begin{array}{cc} A_{m} & \geq \left\lceil \frac{MN\left(4I-N+1\right)-\left(I^{2}+I\right)\left(M+q\right)}{(2MN-M-2)M}\right\rceil , \end{array}$$

*where I=⌊2MN/(M+q)⌋.*

## 3. New Lower Bounds on the Aperiodic Hamming Correlation of Wide-Gap Frequency-Hopping Sequence Sets

In this section, we will generalize the traditional Peng–Fan bound on the aperiodic Hamming correlation for FHSs, and establish two theoretical lower bounds on the maximum aperiodic Hamming correlation for WGFHS sets with given alphabet size, sequence period, and number of sequences.

Below, we first introduce the relevant lemma.

**Lemma** **3** ([7])**.** *Let S be a set of M FHSs with period N over F of size q. For any two FHSs X,Y∈S, denote J=⌊MN/q⌋ and*

(7) $$\begin{array}{c} Q\left(X,Y\right)=\sum_{\tau =0}^{N-1}H_{X,Y}\left(\tau \right). \end{array}$$

*Then, we have*

$$\begin{array}{c} \sum_{X,Y\in S}Q\left(X,Y\right)\geq \frac{M^{2}N^{2}}{q}, \end{array}$$

*and*

$$\begin{array}{c} \sum_{X,Y\in S}Q\left(X,Y\right)\geq (2J+1)MN-(J+1)Iq. \end{array}$$

Next, we will derive two lower bounds on the maximum aperiodic Hamming correlation for WGFHS sets.

**Theorem** **1.** *Let S be an $\left(N,M,q;A_{m}\right)$-WGFHS set with minimum gap D, then*

(8) $$\begin{array}{c} A_{m}\geq \left\lceil \frac{(3MN-Nq+M+q)N}{\left(2MN-M-5)(M+q\right)}\right\rceil , \end{array}$$

*and*

(9) $$\begin{array}{c} A_{m}\geq \left\lceil \frac{\left(4I-N+3)MN-I(I+1)(M+q\right)}{(2MN-M-5)M}\right\rceil , \end{array}$$

*where $I=\left\lfloor 2MN/(M+q)\right\rfloor$.*

**Proof.**  Assume that the alphabet, denoted by $\bar{F}$, is obtained by adding *M* distinct frequencies $f_{q},f_{q+1},\cdots ,f_{q+M-1}$ to the alphabet F of size *q*, and satisfies the following conditions:

(1) For any $f_{i}\in F,\;\left(0\leq i\leq q-1\right)$ and any $f_{j}\in \bar{F},\;\left(q\leq j\leq q+M-1\right)$, we have $f_{j}-f_{i}\geq D$;
(2) For any $f_{j}\in \bar{F},\;\left(q\leq j\leq q+M-2\right)$, we have $|f_{j+1}-f_{j}|\geq D$ and $|f_{q+M-1}-f_{q}|\geq D$.

For any WGFHS $x_{i}=\left(x_{i}\left(0\right),x_{i}\left(1\right),\cdots ,x_{i}\left(N-1\right)\right)$ in $S=\{x_{i}|0\leq i\leq M-1\}$, let $\bar{x_{i}}$ denote the FHS obtained by appending *N* consecutive frequencies selected from $\bar{F}$ to the right of $x_{i}$, that is,

$$\begin{array}{cc} \bar{x_{0}} & =\left(x_{0}\left(0\right),x_{0}\left(1\right),\cdots ,x_{0}\left(N-1\right),f_{q},f_{q+1},\cdots ,f_{q+N-1}\right),\\ \bar{x_{1}} & =\left(x_{1}\left(0\right),x_{1}\left(1\right),\cdots ,x_{1}\left(N-1\right),f_{q+1},f_{q+2},\cdots ,f_{q+N}\right),\\ \; & \;\;\vdots \\ \bar{x_{M-2}} & =\left(x_{M-2}\left(0\right),x_{M-2}\left(1\right),\cdots ,x_{M-2}\left(N-1\right),f_{q+M-2},f_{q+M-1},\cdots ,f_{q+N-3}\right),\\ \bar{x_{M-1}} & =\left(x_{M-1}\left(0\right),x_{M-1}\left(1\right),\cdots ,x_{M-1}\left(N-1\right),f_{q+M-1},f_{q},\cdots ,f_{q+N-2}\right). \end{array}$$

Let $\bar{S}=\left(\bar{x_{0}},\bar{x_{1}},\cdots ,\bar{x_{M-1}}\right)$, then $\bar{S}$ denotes a set consisting of *M* FHSs with period 2N over an alphabet $\bar{F}$ of size q+M. For any two distinct FHSs $\bar{x_{i}}$ and $\bar{x_{j}}$ in $\bar{S}$ with i≠j, according to Definitions 1 and 2, we have

$$\begin{array}{c} H_{\bar{x_{i}},\bar{x_{i}}}\left(\tau \right)=\left\{\begin{array}{cc} A_{x_{i},x_{i}}\left(\tau \right)+N-\tau , & 0\leq \tau \leq N-1,\\ \;\;\;\;\;\;\;\;\;\;\;\;\;\;\;\;0, & \tau =N,\\ A_{x_{i},x_{i}}\left(2N-\tau \right)+\tau -N, & N+1\leq \tau \leq 2N-1. \end{array}\right. \end{array}$$

and

$$\begin{array}{c} H_{\bar{x_{i}},\bar{x_{j}}}\left(\tau \right)=\left\{\begin{array}{cc} A_{x_{i},x_{j}}\left(\tau \right), & 0\leq \tau \leq N-1,\\ \;\;\;\;\;\;\;0, & \tau =N,\\ A_{x_{j},x_{i}}\left(2N-\tau \right), & N+1\leq \tau \leq 2N-1. \end{array}\right. \end{array}$$

Then,

(10) $$\begin{array}{cc} & \sum_{\bar{x_{i}},\bar{x_{j}}\in \bar{S}}\sum_{\tau =0}^{2N-1}H_{\bar{x_{i}},\bar{x_{j}}}\left(\tau \right)\\ = & \sum_{\bar{x_{i}}\in \bar{S}}\sum_{\tau =0}^{2N-1}H_{\bar{x_{i}},\bar{x_{i}}}\left(\tau \right)+\sum_{\begin{array}{c} \bar{x_{i}},\bar{x_{j}}\in \bar{S}\\ \bar{x_{i}}\neq \bar{x_{j}} \end{array}}\sum_{\tau =0}^{2N-1}H_{\bar{x_{i}},\bar{x_{j}}}\left(\tau \right)\\ = & \sum_{\bar{x_{i}}\in \bar{S}}\left[H_{\bar{x_{i}}}\left(0\right)+H_{\bar{x_{i}}}\left(1\right)+H_{\bar{x_{i}}}\left(N-1\right)+\sum_{\tau =2}^{N-2}H_{\bar{x_{i}}}\left(\tau \right)\right]+\\ & \sum_{\bar{x_{i}}\in \bar{S}}\left[H_{\bar{x_{i}}}\left(N\right)+H_{\bar{x_{i}}}\left(N+1\right)+H_{\bar{x_{i}}}\left(2N-1\right)+\sum_{\tau =N+2}^{2N-2}H_{\bar{x_{i}}}\left(\tau \right)\right]+\\ \; & \sum_{\begin{array}{c} \bar{x_{i}},\bar{x_{j}}\in \bar{S}\\ \bar{x_{i}}\neq \bar{x_{j}} \end{array}}\left[\sum_{\tau =0}^{N-1}H_{\bar{x_{i}},\bar{x_{j}}}\left(\tau \right)+\sum_{\tau =N+1}^{2N-1}H_{\bar{x_{i}},\bar{x_{j}}}\left(\tau \right)\right]\\ = & 2MN+\sum_{x_{i}\in S}\left[\sum_{\tau =2}^{N-2}\left(A_{x_{i}}\left(\tau \right)+N-\tau \right)\right]+\sum_{x_{i}\in S}\left[\sum_{\tau =N+2}^{2N-2}\left(A_{x_{i}}\left(2N-\tau \right)+\tau -N\right)\right]+\\ \; & \sum_{\begin{array}{c} x_{i},x_{j}\in S\\ x_{i}\neq x_{j} \end{array}}\sum_{\tau =0}^{N-1}A_{x_{i},x_{j}}\left(\tau \right)+\sum_{\begin{array}{c} x_{i},x_{j}\in S\\ x_{i}\neq x_{j} \end{array}}\sum_{\tau =N+1}^{2N-1}A_{x_{j},x_{i}}\left(2N-\tau \right)\\ = & 2MN\;+\;\sum_{x_{i}\in S}\left[\sum_{\tau =2}^{N-2}\left(A_{x_{i}}\left(\tau \right)+N-\tau \right)\right]\;+\;\sum_{x_{i}\in S}\left[\sum_{\tau =2}^{N-2}\left(A_{x_{i}}\left(N-\tau \right)+\tau \right)\right]\;+\;\sum_{\begin{array}{c} x_{i},x_{j}\in S\\ x_{i}\neq x_{j} \end{array}}\sum_{\tau =0}^{N-1}A_{x_{i},x_{j}}\left(\tau \right)\\ \; & +\sum_{\begin{array}{c} x_{i},x_{j}\in S\\ x_{i}\neq x_{j} \end{array}}\sum_{\tau =1}^{N-1}A_{x_{j},x_{i}}\left(N-\tau \right)\\ \leq & 2MN+\left(N-3\right)MA_{a}+\frac{(N-3)MN}{2}+\left(N-3\right)MA_{a}+\frac{(N-3)MN}{2}+M\left(M-1\right)NA_{c}+\\ \; & M\left(M-1\right)\left(N-1\right)A_{c}\\ = & \left(N-1\right)MN+2\left(N-3\right)MA_{a}+M\left(M-1\right)\left(2N-1\right)A_{c}. \end{array}$$

According to Lemma 3, we can obtain

(11) $$\begin{array}{c} \sum_{\bar{x_{i}},\bar{x_{j}}\in \bar{S}}\sum_{\tau =0}^{2N-1}H_{\bar{x_{i}},\bar{x_{j}}}\left(\tau \right)\geq \frac{4M^{2}N^{2}}{M+q}, \end{array}$$

and

(12) $$\begin{array}{c} \sum_{\bar{x_{i}},\bar{x_{j}}\in \bar{S}}\sum_{\tau =0}^{2N-1}H_{\bar{x_{i}},\bar{x_{j}}}\left(\tau \right)\geq 2\left(2I+1\right)MN-I\left(I+1\right)\left(M+q\right), \end{array}$$

where I=⌊2MN/(M+q)⌋.

Combining Equations (10)–(12), we have

$$\begin{array}{c} \frac{4M^{2}N^{2}}{M+q}\leq \sum_{\bar{x_{i}},\bar{x_{j}}\in \bar{S}}\sum_{\tau =0}^{2N-1}H_{\bar{x_{i}},\bar{x_{j}}}\left(\tau \right)\leq \left(N-1\right)MN+2\left(N-3\right)MA_{a}+M\left(M-1\right)\left(2N-1\right)A_{c}, \end{array}$$

and

$$\begin{array}{cc} 2(2I+1)MN-I(I+1)(M+q) & \leq \sum_{\bar{x_{i}},\bar{x_{j}}\in \bar{S}}\sum_{\tau =0}^{2N-1}H_{\bar{x_{i}},\bar{x_{j}}}\left(\tau \right)\\ \; & \leq \left(N-1\right)MN+2\left(N-3\right)MA_{a}+M\left(M-1\right)\left(2N-1\right)A_{c}. \end{array}$$

Simplifying the above inequalities yields

$$2\left(N-3\right)\left(M+q\right)A_{a}+\left(M-1\right)\left(2N-1\right)\left(M+q\right)A_{c}\geq \left(3MN-Nq+M+q\right)N,$$

and

$$2\left(N-3\right)MA_{a}+M\left(M-1\right)\left(2N-1\right)A_{c}\geq \left(4I-N+3\right)MN-I\left(I+1\right)\left(M+q\right).$$

Since $A_{m}=\max\left\{A_{a},A_{c}\right\}$, we have

$$A_{m}\geq \frac{(3MN-Nq+M+q)N}{\left(2MN-M-5)(M+q\right)},$$

and

$$A_{m}\geq \frac{\left(4I-N+3)MN-I(I+1)(M+q\right)}{(2MN-M-5)M}.$$

This completes the proof. □

By the bounds above, we naturally define the optimality of an aperiodic WGFHS set.

**Definition** **5.** *A WGFHS set S is said to be optimal with respect to the aperiodic Hamming correlation if its relevant parameters achieve the equal sign of Equation *(8)* or Equation *(9)*.*

## 4. Some Examples of WGFHS Sets with Optimal Aperiodic Hamming Correlation

In this section, we will present some examples of WGFHS sets to illustrate the tightness of the bounds derived in Section 3. Among them, the two bounds given by Equations (8) and (9) are new bounds on the maximum aperiodic Hamming correlation of WGFHS sets.

A natural question is whether these bounds can be achieved under certain FHS parameters, i.e., whether they are optimal. We obtain an affirmative answer by numerically evaluating the WGFHS sets in Table 1 via computer programs. Prior to this, we briefly clarify the notation used herein. “Y” indicates that the corresponding parameters achieve the equal sign of the above new bounds, and “N” indicates that the parameters cannot achieve the equal sign of the above new bounds.

The following examples demonstrate that our bounds in Theorem 1 improve the traditional Peng-Fan aperiodic bounds.

**Example** **1.** *Consider a WGFHS set S={X,Y} with parameters N=5,M=2,q=5,D=1, given by*

X=(0,2,4,1,3),

*and*

Y=(0,3,1,4,2).

*It can be verified that $A_{a}=0$ and $A_{m}=1$. Computing the right side of the Peng-Fan aperiodic bounds in Lemma 1 yields*

$$\begin{array}{cc} \left\lceil \frac{(3MN-Nq-N-M-q)N}{\left(2MN-M-1)(M+q\right)}\right\rceil & =0,\\ \left\lceil \frac{\left(4I-N-1)MN-I(I+1)(M+q\right)}{(2MN-M-1)M}\right\rceil & =0. \end{array}$$

*Similarly, computing the right side of the bounds in Lemma 2, we have*

$$\begin{array}{cc} \left\lceil \frac{3MN^{2}-MN-qN^{2}-q}{\left(2MN-M-2)(M+q\right)}\right\rceil & =0,\\ \left\lceil \frac{MN\left(4I-N+1\right)-\left(I^{2}+I\right)\left(M+q\right)}{(2MN-M-2)M}\right\rceil & =0. \end{array}$$

*Finally, computing the right side of the new bounds given by Equations *(8)* and *(9)*, we obtain*

$$\begin{array}{cc} \left\lceil \frac{(3MN-Nq+M+q)N}{\left(2MN-M-5)(M+q\right)}\right\rceil & =1,\\ \left\lceil \frac{\left(4I-N+3)MN-I(I+1)(M+q\right)}{(2MN-M-5)M}\right\rceil & =1. \end{array}$$

*It is evident that the given WGFHS set is optimal with respect to the new bounds in Theorem 1, while it is not optimal with respect to the Peng-Fan aperiodic bounds and the aperiodic WGFHSs bounds in Lemma 2.*

In Table 1, we list several parameters of WGFHS sets that are optimal with respect to the maximum aperiodic Hamming correlation.

Clearly, these numerical examples demonstrate that the proposed bounds are tighter than the previous aperiodic bounds for many practical parameter ranges.

**Remark** **1.** *When the parameters of FHSs satisfy that the denominators in Equations *(3)*–*(6)*, *(8)* and *(9)* are all positive (otherwise, these formulas become mathematically meaningless and should not be adopted), we compare the three groups of lower bounds in the following manner. In particular, if the value computed by the above formulas is negative, the corresponding actual aperiodic Hamming correlation value should be set to 0. This is because the aperiodic Hamming correlation essentially represents the number of frequency collisions and cannot be negative.*

*Comparison of the bounds (without I). Observe that 2MN−M−1>2MN−M−2>2MN−M−5>0; the denominators on the right sides of Equations *(3)*, *(5)* and *(8)* are strictly decreasing. Meanwhile, the numerators satisfy*

$$\left[\left(3MN-Nq+M+q\right)N\right]-\left[3MN^{2}-MN-qN^{2}-q\right]=2MN+Nq+q>0,$$

*and*

$$\left[3MN^{2}-MN-qN^{2}-q\right]-\left[\left(3MN-Nq-N-M-q\right)N\right]=N^{2}+q\left(N-1\right)>0.$$

*Hence, along the right sides of Equations *(3)*, *(5)* and *(8)*, the numerators are strictly increasing while the denominators are strictly decreasing; it follows that the right sides of Equations *(3)*, *(5)* and *(8)* are strictly increasing.*

*Comparison of the bounds (containing I). Similarly, the denominators are again strictly decreasing along the right sides of Equations *(4)*, *(6)* and *(9)*. For the numerators,*

$$\left[\left(4I-N+3\right)MN-I\left(I+1\right)\left(M+q\right)\right]-\left[MN\left(4I-N+1\right)-\left(I^{2}+I\right)\left(M+q\right)\right]=2MN>0,$$

*and*

$$\left[MN\left(4I-N+1\right)-\left(I^{2}+I\right)\left(M+q\right)\right]-\left[\left(4I-N-1\right)MN-I\left(I+1\right)\left(M+q\right)\right]=2MN>0.$$

*Again, the numerators increase and the denominators decrease along the right sides of Equations *(4)*, *(6)* and *(9)*, so the right sides of Equations *(4)*, *(6)* and *(9)* are strictly increasing.*

*Therefore, the proposed bounds in Equations *(8)* and *(9)* are strictly tighter than the Peng-Fan aperiodic bounds in Equations *(3)* and *(4)* and the aperiodic WGFHS bounds in Equations *(5)* and *(6)*, for any feasible parameter set as described above.*

## 5. Conclusions

In this paper, we derived two lower bounds on the aperiodic Hamming correlation of WGFHS sets. Extensive numerical examples verify that the proposed lower bounds are tighter than the existing aperiodic bounds for both FHSs and WGFHSs. These results not only enrich the theoretical framework of WGFHSs but also provide theoretical performance benchmarks for practical anti-jamming FH system design.

One limitation of the present work is that the resulting expressions do not explicitly involve the minimum gap parameter *D*, despite the fact that the wide-gap condition corresponding to parameter *D* serves as an essential prerequisite throughout the proof. In the final closed-form expressions presented in the paper, such dependence is incorporated only via the worst-case counting argument. Therefore, future research directions include deriving bounds that explicitly depend on *D*, as well as constructing infinite families of WGFHS sets attaining our newly derived bounds. Explicit algebraic constructions for optimal WGFHS sets under the aperiodic Hamming correlation criterion remain an interesting open problem worthy of further investigation.

## Figures and Tables

**Table 1:** Some optimal WGFHS sets for aperiodic Hamming correlation.

(N,M,q,D)	$A_{a}$	$A_{m}$	(3) or (4)	(5) or (6)	(8) or (9)
(5, 2, 5, 1)	0	1	N	N	Y
(6, 20, 5, 1)	1	1	N	Y	Y
(7, 20, 5, 1)	1	1	N	Y	Y
(8, 19, 5, 1)	1	1	Y	Y	Y
(9, 19, 5, 1)	1	1	Y	Y	Y
(6, 20, 6, 1)	0	1	N	N	Y
(7, 798, 6, 1)	1	1	N	Y	Y
(8, 1048, 6, 1)	1	1	N	Y	Y
(7, 9836, 7, 1)	0	1	N	Y	Y

## Data Availability

Data are contained within the article.
